# Proctotomy leak following laparoscopic total gastrectomy with transrectal specimen extraction for gastric cancer: a case report

**DOI:** 10.1186/s12893-021-01217-z

**Published:** 2021-04-27

**Authors:** Haipeng Meng, Jinchao Liu, Hui Xu, Song Wang, Yu Rong, Yanling Xu, Gang Yu

**Affiliations:** 1 Department of General Surgery, Cheeloo College of Medicine, Qilu Hospital (Qingdao), Shandong University, 758 Hefei Road, 266035 Qingdao, Shandong China; 2 Department of Gastrointestinal Surgery, Zibo Municipal Hospital, 255400 Zibo, Shandong China; 3 Department of Anesthesiology, Zibo Municipal Hospital, 255400 Zibo, Shandong China; 4 Department of Respiratory Medicine, Cheeloo College of Medicine, Qilu Hospital (Qingdao), Shandong University, 758 Hefei Road, 266035 Qingdao, Shandong China

**Keywords:** Gastric cancer, Laparoscopic gastrectomy, Natural orifice specimen extraction, Proctotomy leak, Case report

## Abstract

**Background:**

Despite increasing acceptance in colorectal surgery, natural orifice specimen extraction (NOSE) surgery for the treatment of gastric cancer is still in its infancy, especially via the transrectal approach, which was barely reported. So little is known about its complications. Here we report the first case of proctotomy leak after transrectal NOSE gastrectomy, and our experience in preventive interventions.

**Case presentation:**

A 62-year-old male patient complaining of upper abdominal pain who underwent open distal gastrectomy for gastric cancer one year ago was diagnosed with recurrent gastric cancer by gastroscopic biopsy. We performed laparoscopic total gastrectomy with transrectal specimen extraction on the patient. The operation was completed in a total laparoscopic approach and the specimen was extracted through a 3 cm longitudinal incision in the anterior wall of the upper rectum, then interrupted sutures were used for full-thickness closure of the rectal incision. The operative time was 470 min and intra-operative blood loss was 100 mL. The postoperative pathological examination showed pT1bN0M0 gastric adenocarcinoma. The patient developed proctotomy leak on the 10th postoperative day. We analyzed the causes of this rare complication and put forward a series of technical improvements. After failure of conservative treatment, a diverting ileostomy was created and the patient eventually recovered. We successfully prevented proctotomy leak in the subsequent 20 transrectal NOSE gastrectomies using improved techniques.

**Conclusions:**

Proctotomy leak after transrectal specimen extraction should be considered among the complications of NOSE surgery and can be prevented by technical precautions.

## Background

Natural orifice specimen extraction (NOSE) surgery has attracted world-wide attention because of its great advantages including minimal abdominal trauma and postoperative pain, fast recovery, avoidance of wound-related complications and cosmetic effect [1]. While the use of transvaginal NOSE gastrectomy has been frequently reported [2], transrectal cases are rare. Likewise, complications exclusive to use of transvaginal route including rectovaginal fistula, dyspareunia and vaginal cancer recurrence have been reported [3], but no literature exists regarding the exclusive complication of transrectal specimen extraction, e.g. proctotomy leak. This study reports a rare and challenging case of proctotomy leak after transrectal NOSE gastrectomy, and discusses the etiology and precautions.

## Case presentation

A 62-year-old male patient received open radical gastrectomy with a Billroth I reconstruction for distal gastric cancer in another hospital. One year after the primary operation, he underwent gastroscopic examination due to complaint of upper abdominal pain lasting for one month. A 1.5 cm × 1.5 cm mass was discovered at the gastroduodenal anastomosis, which was confirmed by pathological biopsy as a gastric adenocarcinoma. There was no metastasis on radiological screening (clinical stage: cT1bN0M0). Then the patient was referred to our hospital for treatment of recurrent gastric caner. He has no comorbidities and his body mass index (BMI) was 31.0 kg/m^2^. He had a history of smoking and drinking for more than 30 years. There was no significant family history.

The patient was operated under general anesthesia as American Society of Anesthesiologists Grade 1. He was placed in the functional lithotomy position and the conventional V glyph 5-port method was used to set the trocars [4]. Radical total gastrectomy was performed in a complete laparoscopic approach. Upon resection, the specimen was transferred to the pelvic cavity for extraction later. Then an intracorporeal Roux-en-Y digestive tract reconstruction was completed, in which the esophagojejunostomy was handsewn.

The specimen extraction procedure was as follows. The anus was sufficiently dilated and the rectum was extensively rinsed with iodine water. Electrocautery was used to cut the anterior wall of the upper rectum just above the peritoneal reflection longitudinally by 3 cm in length. A sterile plastic sleeve was introduced into the peritoneal cavity through the anus. The specimen was grasped with an ovary clamp through the anus-rectum and pulled out of the body. After removal of the plastic sleeve, the rectal incision was repeatedly disinfected with iodophor gauze, and then interrupted sutures were used for full-thickness closure of the incision. The operation was completed after irrigation of the pelvic cavity and rectal lumen with iodine water.

The operative time was 470 min and intra-operative blood loss was 100 mL. The postoperative pathological examination showed T1bN0M0 gastric adenocarcinoma. Postoperatively, the patient followed the same protocol of nutritional support with the standard total gastrectomy patients. He was allowed to take sips of water on the first postoperative day. A fluid diet was introduced on postoperative day 3, and he was allowed to eat soft bread 5 days postoperatively.

**Fig. 1:**
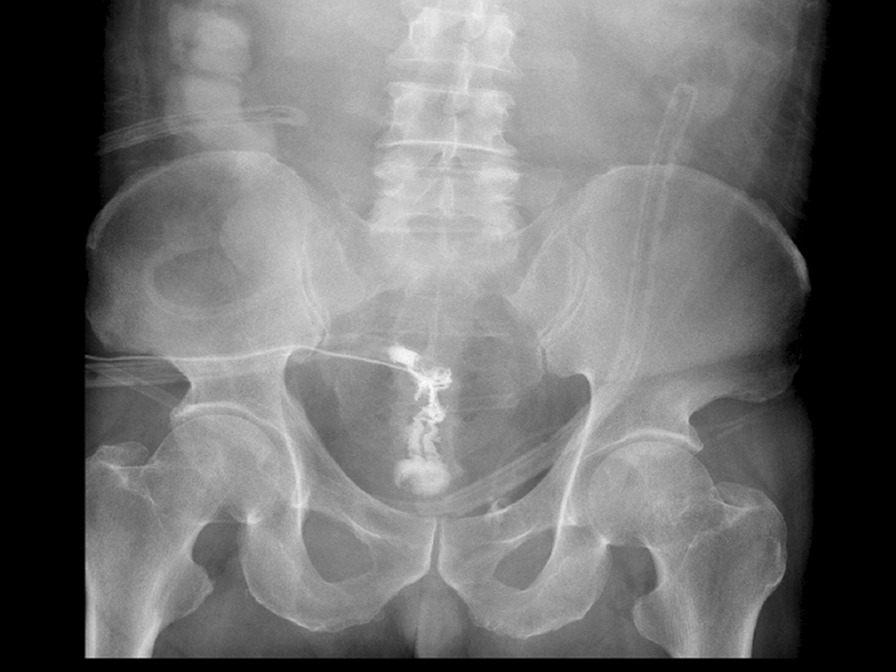
Ioversol leaks from the pelvic drainage tube to the rectal cavity

**Fig. 2:**
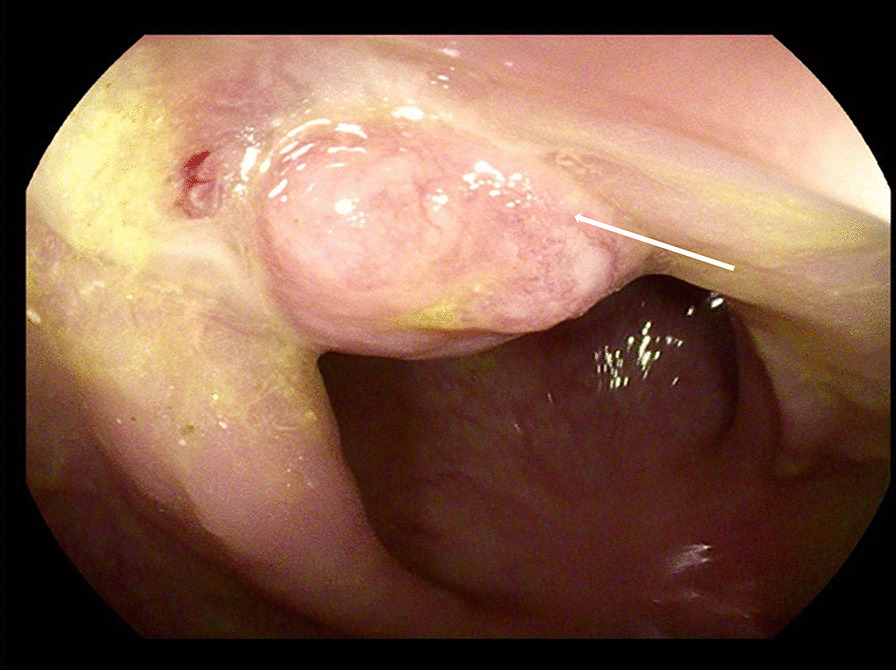
Colonoscopy shows healed proctotomy leak (arrow) with no stenosis in the rectal cavity

Ten days after the operation, the patient presented with sudden onset of abdominal pain, accompanied by fever, chills and sweating. Physical examination revealed tenderness in the lower abdomen with rebound pain. Meanwhile, 200 ml turbid fluid drained from the pelvic drainage tube. We injected ioversol into the pelvic drainage tube under X-ray and found it leaking into the rectal lumen (Fig. 1), thus confirmed the diagnosis of proctotomy leak. After nine days of conservative treatment including adequate pelvic irrigation and drainage, parenteral nutrition and antibiotics administration, the leak persisted and the intra-abdominal infection deteriorated, so a diverting ileostomy was performed. Then the patient gradually recovered and was discharged from hospital. Colonoscopy was performed 6 months later, revealing healed proctotomy leak with only a streak of mucosal hyperplasia and edema (Fig. 2); no stenosis or scar contracture was present in the rectal lumen. So the ileostomy was closed. After 3 years of follow up, no tumor recurrence or anal dysfunction was detected.

## Discussion and conclusions

We analyzed the etiology of proctotomy leak and thought the following reasons were of utmost importance. First, the incision in the anterior wall of the rectum was too short (3 cm) to remove the specimen (the maximum diameter was 5 cm) smoothly despite the malleability of the rectal wall. The patient was obese and the omentum was thick, leading to a relatively large specimen for transrectal extraction. Violent withdrawal led to rupture of the rectal wall. Second, the rectal incision was too low, just above the peritoneal reflection, so the rupture of the rectal wall went below the peritoneal reflection, making it difficult to be repaired. Third, we failed to make a straight rectal incision because of the flexure of rectum, making it difficult to be closed. Fourth, interrupted sutures might be insufficient to close the rectal incision, as under laparoscopic vision it was difficult to perceive the distance between stitches precisely, sometimes it maybe too large. Fifth, the water injection and air inflation methods were not used to check if the rectal incision was completely closed.

**Fig. 3:**
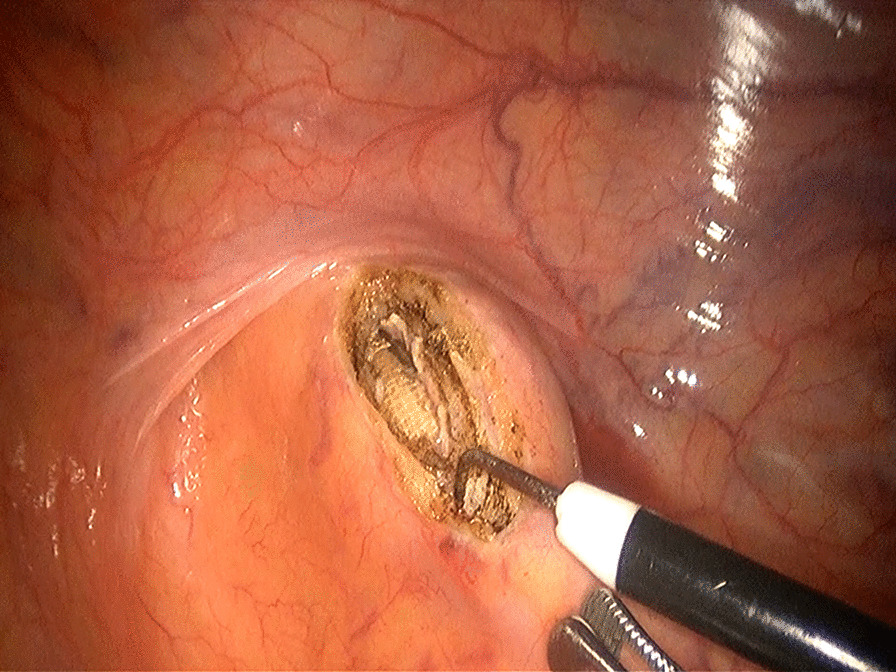
An incision is made in the anterior wall of the upper rectum

**Fig. 4:**
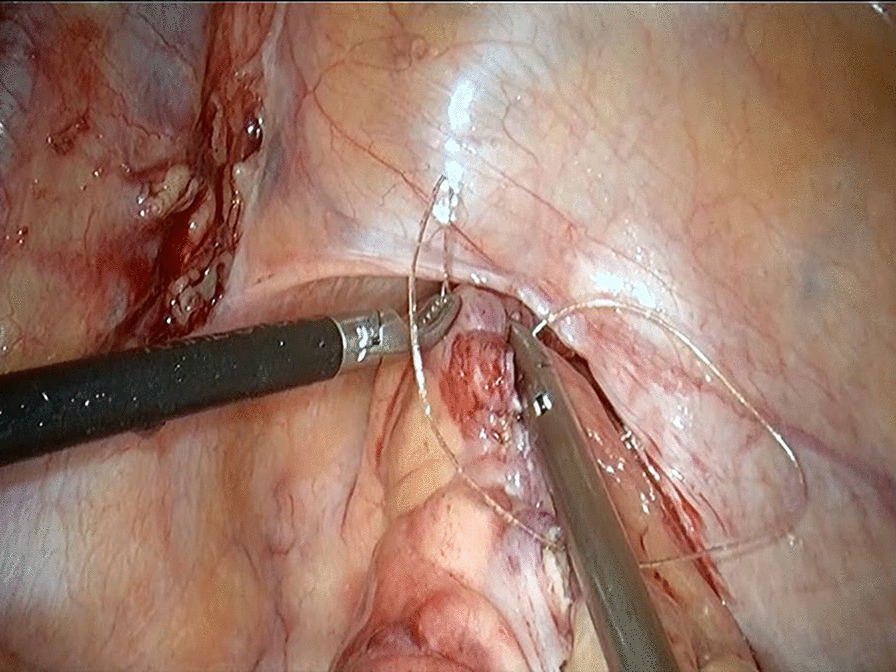
The rectal incision is closed with a continuous full-thickness suture

**Fig. 5:**
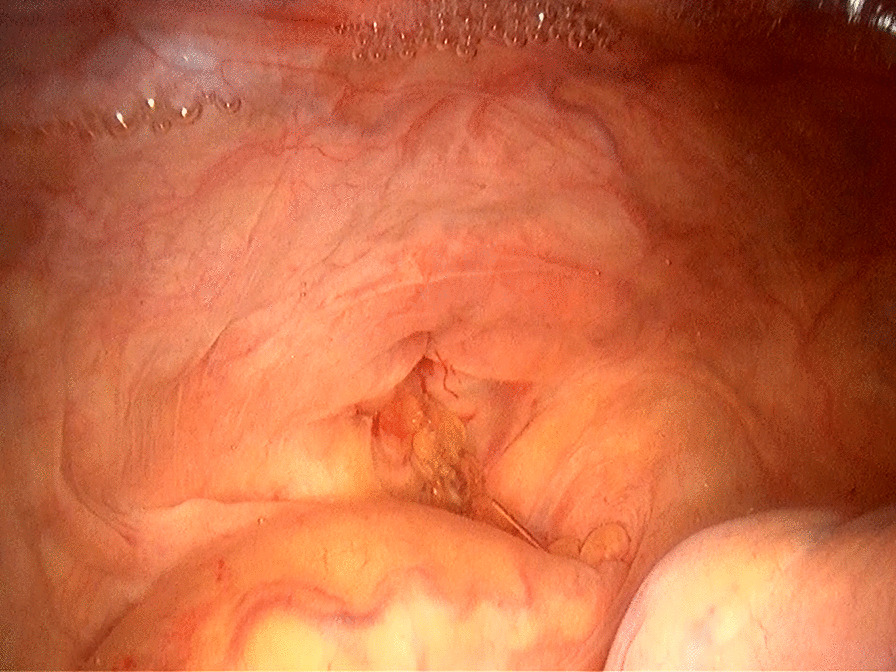
An air inflation test is used to check the completeness of the sutured incision

Based on the aforementioned analysis, we improved the procedure in five aspects. First, strict indications are implemented for transrectal specimen extraction. The maximum diameter of specimen should be ≤ 4 cm and the patient’s BMI ≤ 28 kg/m^2^. Second, the upper rectum should be pulled straight before being incised and the longitudinal incision should locate in the midline of the anterior wall (Fig. 3). Third, the incision is recommended to be 5 cm above the peritoneal reflection (Fig. 3) and large enough for smooth specimen extraction [5]. Fourth, for closing the rectum longitudinally, a barbed wire suture should be made from the distal end to the proximal end with a continuous full-thickness suture, followed by inversion of the suture line with another round of full-thickness stitches which locate between the former round of stitches to guarantee a complete closure (Fig. 4) [6]. Fifth, after stitching, an air test is required to detect whether the sutured incision is intact (Fig. 5). We followed these measures in our subsequent 20 transrectal NOSE gastrectomies and no more proctotomy leak occurred.

There are three potential natural orifices for extraction of gastrectomy specimen: the mouth, vagina and rectum/anus. It has been reported that surgical specimen of early gastric cancer can be removed transorally, but the specimen needs to be segmented into pieces, which poses the risk of tumor dissemination [7]. Considering malleability and safety, vagina is the most ideal route for specimen extraction, which is most frequently used [8]. But for male patients, the rectum is the only appropriate route for gastric NOSE surgery [5]. However, transrectal specimen extraction poses risks of pelvic contamination and tumor dissemination. In order to solve these problems, we recommend that only early gastric cancer and relatively small tumor be considered for this procedure, and that preventive measures such as prophylactic antibiotics administration, mechanical bowel preparation, thorough rectal disinfection, use of a sterile plastic sleeve and pelvic irrigation be implemented.

In conclusion, it is necessary to take precautions for proctotomy leak in transrectal NOSE surgery. We believe that this complication will be prevented by measures now routinely undertaken in our hospital, making transrectal NOSE surgery safe and feasible.

## Data Availability

All data generated or analyzed during this study are included in this published article.

## Abbreviations

NOSE: Natural orifice specimen extraction
BMI: Body Mass Index
